# Screening of an FDA-Approved Library for Novel Drugs against *Y. pestis*

**DOI:** 10.3390/antibiotics10010040

**Published:** 2021-01-03

**Authors:** David Gur, Theodor Chitlaru, Emanuelle Mamroud, Ayelet Zauberman

**Affiliations:** Department of Biochemistry and Molecular Genetics, Israel Institute for Biological Research, 24 Reuven Lerer, Ness Ziona 74010001, Israel; gurd@iibr.gov.il (D.G.); theodorc@iibr.gov.il (T.C.); emmym@iibr.gov.il (E.M.)

**Keywords:** *Y. pestis*, plague, repurposing, antibacterial, antibiotic resistance, FDA-approved drugs

## Abstract

*Yersinia pestis* is a Gram-negative pathogen that causes plague, a devastating disease that kills millions worldwide. Although plague is efficiently treatable by recommended antibiotics, the time of antibiotic therapy initiation is critical, as high mortality rates have been observed if treatment is delayed for longer than 24 h after symptom onset. To overcome the emergence of antibiotic resistant strains, we attempted a systematic screening of Food and Drug Administration (FDA)-approved drugs to identify alternative compounds which may possess antibacterial activity against *Y. pestis*. Here, we describe a drug-repurposing approach, which led to the identification of two antibiotic-like activities of the anticancer drugs bleomycin sulfate and streptozocin that have the potential for designing novel antiplague therapy approaches. The inhibitory characteristics of these two drugs were further addressed as well as their efficiency in affecting the growth of *Y. pestis* strains resistant to doxycycline and ciprofloxacin, antibiotics recommended for plague treatment.

## 1. Introduction

*Yersinia pestis* is a Gram-negative pathogen that causes plague, a devastating disease that has killed millions throughout human history. The two main forms of the disease are the bubonic and the pneumonic plague. Bubonic plague develops following transmission of the pathogen to humans by infected fleas. Primary pneumonic plague results from the inhalation of bacteria-containing droplets or aerosols and is capable of spreading from person to person [1], leading to the rapid progression of a contagious and fatal disease. Left untreated, it usually leads to death within 3–4 days after exposure [2]. Most recently, the 2017 epidemic of *Y. pestis* infection on the island of Madagascar had an unusual predominance of pneumonic cases (1878 out of 2414 clinically suspected plague cases), and strict infection and prevention control measures had to be taken to stop the propagation of the infection [3]. Plague is treatable by recommended antibiotics; however, high mortality rates have been observed if treatment is delayed [4]. The antibiotics recommended to treat plague include aminoglycosides, fluoroquinolones, tetracyclines, and chloramphenicol [2]. However, the isolation of naturally occurring antibiotic-resistant *Y. pestis* strains [5,6] emphasizes the urgent need for the development of additional countermeasures. In addition, the possibility that such strains may be maliciously used as bioweapons represents a high-priority public health concern.

In recent years, health officials have warned that antimicrobial resistance is a major threat to global health. In response, efforts are focusing on exploring the potential use of existing drugs, an approach known as “drug repurposing” [7,8]. Finding new uses for existing drugs can be achieved either on the basis of a detailed understanding of the chemistry of individual compounds and their potential targets from in silico modeling followed by in vitro cell culture and in vivo animal studies or by directly screening a large library of compounds in disease-relevant phenotypic assays, thus allowing an unbiased approach to obtain successful drug candidates.

In this study, we screened 1363 compounds of an FDA-approved drug library (APExBIO DiscoveryProbe) for their ability to reduce the growth of *Y. pestis* and identified a couple of candidates that exhibited potent activity against *Y. pestis* growth in vitro, suggesting that they may potentially be used to treat plague infection.

## 2. Results and Discussion

### 2.1. Design of the Study

In this study, we employed the APExBIO DiscoveryProbe library of FDA-approved drugs (https://www.apexbt.com/discoveryprobetm-fda-approved-drug-library.html) to screen for inhibitory molecules that reduce the growth of *Y. pestis* and may serve for designing novel antiplague therapeutic strategies. The library included 1363 compounds belonging to a variety of drug classes, not only those known for the treatment of infectious diseases, such as antibiotics, but also groups of drugs recommended for the treatment of noninfectious pathological conditions, such as cancer, cardiovascular and metabolic diseases. According to the rationale of the survey, compounds were distinguished as potential inhibitors and selected for subsequent interrogation if they significantly affected the in vitro bacterial growth, as manifested by a reduced turbidity of the culture in comparison to that of untreated bacteria (see Section 2.2). For compound screening, *Y. pestis* bacteria were cultured in 96-well microtiter plates at 37 °C, conditions considered to mimic those encountered in the mammalian host. In preliminary experiments, heart infusion broth (HIB) medium was used to afford significantly superior growth of *Y. pestis* compared to that in the standard medium Mueller−Hinton broth (MHB), as stipulated by the clinical and laboratory standards institute [9] for antimicrobial susceptibility assays, and was therefore used for the screening processes.

### 2.2. Identification of Novel Potential Antiplague Drugs

All compounds were first tested at a concentration of 10 µM, and the potential candidate drugs considered were those that exhibited a ten-fold or higher inhibition index (90% reduction of bacterial growth compared to that of untreated bacteria) after 24 h and a five-fold or higher inhibition index (80% reduction) after 48 h. Indeed, in some cases, after 48 h, the inhibitory effect of some of the putative drugs may have occurred due to their degradation in the conditions employed for screening. Treatment with the positive control antibiotic ciprofloxacin (0.05 µg/mL) resulted in total inhibition of bacterial growth. Fifty-five compounds meeting the inhibitory criterion were selected after the primary screen and retested in a secondary screen, resulting in confirmation of 45 compounds exhibiting an inhibitory effect on *Y. pestis* growth (3.3% confirmation rate for the 1363 compounds). These 45 compounds, depicted in Table 1, included 36 antibiotics that are known to inhibit *Y. pestis* or belong to categories of drugs known to possess antimicrobial activity against *Y. pestis*. Mitomycin C, in spite of its observed inhibitory effect, was excluded from the subsequent analysis due to its reported phage induction ability, which may have complicated the interpretation of the results [10,11]. Eight additional drugs (out of 45) have not been previously reported as potential growth inhibitory agents against *Y. pestis*. Based on their previously known clinical indications, the novel antiplague compounds (Table 2) included (i) the three antiseptic drugs cetrimonium bromide, cetylpyridinium chloride and chlorquinaldol, (ii) the two antiparasite (vermifuge) drugs closantel and closantel sodium, (iii) the two anticancer drugs streptozocin and bleomycin sulfate, and (iv) the antifungal drug zinc pyrithione.

### 2.3. Characterization of the Anti-Y. pestis Inhibitory Activity of Novel Drugs

**Figure 1:**
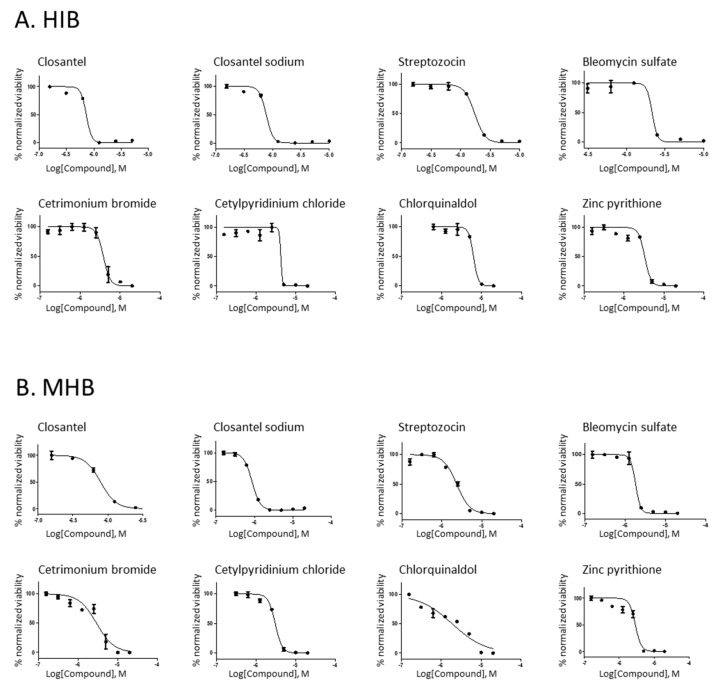
Concentration response curves of the inhibition of *Y. pestis* growth by eight nonantibiotic drugs. Eight selected compounds were tested in 8-fold dilutions ranging from 20 µM to 0.15 µM. The anti-*Y. pestis* activity of the selected drugs was confirmed for closantel, closantel sodium, streptozocin, bleomycin sulfate, cetrimonium bromide, cetylpyridinium chloride, chlorquinaldol and zinc pyrithione grown in (**A**) heart infusion broth (HIB) or (**B**) Mueller−Hinton broth (MHB). Data points represent the mean, and error bars represent the SEM; *n* = 3.

The eight novel, potentially antiplague drugs were further interrogated for in vitro efficacy by determining their *Y. pestis* growth inhibitory concentration 50% (IC_50_) and inhibitory concentration 90% (IC_90_). These values were quantified in HIB and in MHB according to the CLSI guidelines (Figure 1 and Table 2). In addition, their minimal inhibitory concentration (MIC) were calculated in HIB (Table 2).

Six of the eight drugs were not further investigated because they were either used in the past solely for veterinary purposes (closantel and closantel sodium) or reportedly employed for antiseptic external therapy only (cetrimonium bromide, cetylpyridinium chloride, chlorquinaldol and zinc pyrithione). The two anticancer compounds bleomycin sulfate and streptozocin, which were previously reported to serve as human treatments, were selected for further analysis. Notably, both bleomycin and streptozocin were shown to efficiently inhibit the growth of other Gram-positive and Gram-negative bacteria, including *Staphylococcus epidermidis*, *Klebsiella pneumonia* and *Escherichia coli* [12,13].

Bleomycin is a radiomimetic compound which forms complexes with iron that reduce molecular oxygen to superoxide and hydroxyl radicals inflicting DNA damage (single and double-strand breaks) that results in preferential inhibition of DNA synthesis in cancer cells [14,15]. This compound is used to treat hematologic (Hodgkin’s and non-Hodgkin’s lymphomas) as well as solid malignancies (testicular, ovarian and cervical cancers). The standard dose of bleomycin (adjusted based on body surface area, BSA) for the treatment of cancer patients is 15 mg/m^2^ (intravenous administration), resulting in a reported circulatory concentration (Cmax) of 706 µM and exhibiting a circulatory half-life (T1/2) of 4 h [16]. This bleomycin dose is more than two orders of magnitude higher than the IC_90_ of 3.77 µM observed for inhibition of *Y. pestis* growth (Table 2), suggesting that an effective anti-*Y pestis* treatment with bleomycin may be well tolerated. Streptozocin is an alkylating antineoplastic agent which inhibits synthesis of DNA both in microorganisms and mammalian cells by cross-linking the DNA strands [17]. It is used to treat metastatic cancer of pancreatic islet cells. The recommended dose for oncologic treatment is 1.5 g/m^2^ by intravenous administration, resulting in an effective circulatory concentration (Cmax) of 1438 µM with a circulatory T1/2 of 0.22 h [16]. This concentration is 300 times higher than the concentration required for inhibition of *Y. pestis* growth (IC_90_ = 4.75 µM, Table 2), suggesting, as in the case of bleomycin, that efficient treatment of *Y. pestis* infection requires well-tolerated doses. Of note, several anticancer compounds possessing DNA-damaging activity (such as lomustine, irinotecan, topotacan and busulfan) which were screened in the current study, were not found to inhibit the growth of *Y. pestis*.

As new drugs are needed against antibiotic-resistant strains, bleomycin sulfate and streptozocin were tested for their ability to inhibit the growth of antibiotic-resistant strains of *Y. pestis*. In view of the fact that ciprofloxacin and doxycycline represent the antibiotics of choice for postexposure prophylaxis and treatment of plague infections in the event of an outbreak, we used nonvirulent *Y. pestis* strains that are resistant to ciprofloxacin and doxycycline [18,19]. These strains are derived from the fully virulent *Y. pestis* Kim53 strain. The IC_90_ calculated for the inhibition of growth promoted by bleomycin sulfate in the ciprofloxacin (3.8 µM) or doxycycline (7.2 µM) resistant strains clearly demonstrated that bleomycin sulfate may effectively prevent the growth of *Y. pestis* antibiotic-resistant strains. Similarly, for streptozocin, the IC_90_ = 4.6 µM for the ciprofloxacin-resistant strain and IC_90_ = 26 µM for the doxycycline-resistant strain indicated that streptozocin may serve as a new candidate to combat drug-resistant *Y. pestis* strains. Of note, the initial screen was conducted under BL2 laboratory conditions using the EV76 attenuated strain (see above). On the basis of previous studies from our laboratory, the results obtained using this strain may be extrapolated to fully virulent strains. While this aspect will require future confirmation, at least in the case of susceptibility to bleomycin and streptozocin, the EV76 and Kim53 derived attenuated-strains are very similar.

Bleomycin and streptozocin, similar to other drugs employed for the treatment of cancer, are cytotoxic, and their administration may be accompanied by severe side effects, although the effective concentration for *Y. pestis* growth inhibition determined in this study appears to be considerably lower than the concentration required for effective cancer chemotherapy. Furthermore, the treatment regimen is expected to be much shorter than that required for cancer treatment. Repurposing anticancer drugs for infectious diseases has been reported despite potential side effects [20]. For example, miltefosine was approved to treat leishmaniasis [21], and the implementation of treatments based on gallium compounds to control *P. aeruginosa* in patients with cystic fibrosis has completed phase II clinical trials [22]. Concerns regarding the clinical use of bleomycin and streptozocin in the treatment of bacterial infections may be mitigated by considering their therapeutic benefit in the case of severe infection, especially those caused by strains resistant to the antibiotics of choice. Such strains resistant to individual antibiotics or exhibiting multidrug resistance have become frequent in recent decades, and treatment options for patients with life-threatening resistant infections have become alarmingly limited [23,24]. Furthermore, the COVID-19 pandemic seems to have rapidly increased the use of antibiotics worldwide [25], accelerating the threat of antimicrobial resistance. Therefore, drugs such as bleomycin sulfate and streptozocin or the other compounds identified in the present study may represent an important basis for designing novel therapeutic strategies. The implementation of the novel drugs for the treatment of plague will involve future studies addressing their in vivo efficiency as well as the possibility of mitigating their possible adverse effects [26]. It is worth noting that previous studies established that bleomycin sulfate inhibits the growth of *Klebsiella pneumonia* at an IC_50_ very similar to the value calculated in the present study for *Y. pestis* [27]. Further studies will address the applicability of the drugs identified in the current survey for the treatment of other infections caused by multidrug-resistant Gram-negative pathogens of public health concern.

## 3. Materials and Methods

### 3.1. Bacterial Strains and Reagents

The *Y. pestis* strains used in this study included the live vaccine strain EV76 (*pgm*-[Girard’s strain]) and two antibiotic-resistant *Y. pestis* strains, ciprofloxacin-resistant strain #66-6 [19] and doxycycline-resistant strain #36-4-18 [18] derived from the avirulent parental pCD1 and pPCP1-cured Kim53 strain [28,29].

### 3.2. Compound Library and Screening

The FDA-approved compound library (L1021) was purchased from APEx-BIO, USA. The library consists of 1363 compounds, which were prepared at 10 mM in DMSO and kept at −70 °C until use. The library compounds were screened at a concentration of 10 µM against the *Y. pestis* EV76 strain to identify antibacterial drugs. *Y. pestis* bacteria were grown on brain heart infusion agar (BHIA, BD, USA, cat. 241830) for 48 h at 28 °C. Bacterial colonies were harvested and diluted in heart infusion broth (HIB, BD, USA, cat. 238400) to an OD_660_ of 0.001 (~5 × 10^5^ cfu/mL) and grown in 96-well plates at 37 °C in HIB or Mueller−Hinton broth (MHB, BD, USA, 212322) for 24 and 48 h. At the end of the incubation period, bacterial growth was assessed by measuring the optical density at 630 nm using a Synergy H1 Microplate Reader (BioTek Instruments, Winooski, VT, USA). As a positive control for growth inhibition, bacteria were exposed to ciprofloxacin (Teva, Israel, SH270715) at a concentration of 0.05 µg/mL, which is equivalent to 4 × the minimal inhibitory concentration (MIC) for the EV76 strain. Notably, the OD_630_ of bacteria in HIB-containing ciprofloxacin was similar to that of HIB medium alone that served as a blank.

### 3.3. Statistics and Data Analysis

Raw plate reads were first normalized to those of relative controls. Bacterial growth in HIB medium was considered 100% viability, and HIB medium alone was considered 0% viability. Concentration-response curves were determined for IC_50_ and IC_90_ calculations using the GraphPad Prism 5 statistical package. The minimal inhibitory concentrations (MIC) were calculated on the basis of the growth of the bacteria in HIB for 24 h at 37 °C in the presence of serially diluted antibacterial compound.

## 4. Conclusions

In summary, the two FDA-approved drugs bleomycin sulfate and streptozocin have potent activity against antibiotic-resistant *Y. pestis* bacteria growing in vitro and may serve as new antibacterial drugs to treat plague infection.

## Figures and Tables

**Table 1:** Compounds inhibiting *Y. pestis* growth.

No	CAS Number ^a^	Drug Name	Primary Action ^b^	% Growth ^c^	
				24 h	48 h
1	112811-59-3	Gatifloxacin	Fluoroquinolone antibiotic	<0.1	<0.1
2	364622-82-2	Doripenem hydrate	Carbapenem antibiotic	<0.1	<0.1
3	69-52-3	Ampicillin	β-lactam antibiotic	<0.1	<0.1
4	64-75-5	Tetracycline hydrochloride	Antibiotic	<0.1	<0.1
5	161715-24-8	Tebipen pivoxil	Carbapenem antibiotic	<0.1	4
6	148016-81-3	Doripenem	Carbapenem antibiotic	<0.1	7
7	119478-56-7	Meropenem trihydrate	β-lactam antibiotic	5	7
8	26787-78-0	Amoxicillin	β-lactam antibiotic	<0.1	5
9	7177-48-2	Ampicillin trihydrate	β-lactam antibiotic	4	6
10	69-57-8	Penicillin G sodium	β-lactam antibiotic	3	6
11	104376-79-6	Ceftriaxone sodium trihydrate	cephalosporin antibiotic	<0.1	<0.1
12	57-09-0	Cetrimonium bromide (CTAB)	antiseptic	1	4
13	123-03-5	Cetylpyridinium chloride	Anti-infection antiseptic	<0.1	2
14	72-80-0	Chlorquinaldol	Antifungal and antibacterial	<0.1	16
15	57808-65-8	Closantel	Gram-positive antibacterial activity inhibitor	5	15
16	61438-64-0	Closantel sodium	Gram-positive antibacterial activity inhibitor	3	8
17	73231-34-2	Florfenicol	Antibacterial agent	4	6
18	98079-52-8	Lomefloxacin HCl	Fluoroquinolone antibiotic	7	11
19	56391-57-2	Netilmicin sulfate	Active aminoglycoside antibiotic	9	7
20	59703-84-3	Piperacillin sodium	Semisynthetic, broad-spectrum, ampicillin derived ureidopenicillin antibiotic	<0.1	5
21	32986-56-4	Tobramycin	Aminoglycoside antibiotic	4	7
22	37091-65-9	Azlocillin sodium salt	Semisynthetic penicillin and β-lactam antibiotic	7	9
23	124858-35-1	Nadifloxacin	Topical fluoroquinolone antibiotic	4	10
24	70458-95-6	Pefloxacin mesylate	Synthetic chemotherapeutic and antibacterial agent	4	9
25	13292-46-1	Rifampin	DNA-dependent RNA polymerase inhibitor antibiotic	4	11
26	24390-14-5	Doxycycline hyclate	tetracycline-class antibiotic	<0.1	<0.1
27	1950-7-7	Mitomycin C	Inhibits DNA synthesis, antibiotic, antitumor agent	<0.1	<0.1
28	18883-66-4	Streptozocin	Antibiotic, antitumor agent	<0.1	1.4
29	186826-86-8	Moxifloxacin HCl	Fluoroquinolone antibiotic	<0.1	<0.1
30	115550-35-1	Marbofloxacin	Fluoroquinolone antibiotic for veterinary use	<0.1	<0.1
31	9041-93-4	Bleomycin Sulfate	Chemotherapy agent, induces DNA strand break	<0.1	<0.1
32	91832-40-5	Cefdinir	Third-generation cephalosporin antibiotic	<0.1	2
33	62893-19-0	Cefoperazone	Third-generation cephalosporin antibiotic	<0.1	2
34	98106-17-3	Difloxacin HCl	Quinolone antimicrobial antibiotic	<0.1	1
35	738-70-5	Trimethoprim	Bacteriostatic antibiotic	9	14
36	61379-65-5	Rifapentine	Antibiotic drug used in the treatment of tuberculosis	7	4
37	163253-35-8	Sitafloxacin hydrate	Broad-spectrum oral fluoroquinolone antibiotic	4	3
38	13463-41-7	Zinc pyrithione	Proton pump inhibitor	2	7
39	82419-36-1	Ofloxacin	Fluoroquinolone antibiotic	8	10
40	10592-13-9	Doxycycline HCl	Tetracycline antibiotic	5	7
41	93106-60-6	Enrofloxacin	Fluoroquinolone antibiotic	3	8
42	64485-93-4	Cefotaxime (sodium salt)	Cephalosporin antibiotic	7	9
43	70458-96-7	Norfloxacin	Fluoroquinolone antibiotic	9	9
44	3963-95-9	Methacycline HCl	Tetracycline antibiotic	0	7
45	64953-12-4	Moxalactam (sodium salt)	β-lactam antibiotic	<0.1	<0.1

^a^ Registry number according to the Chemical Abstract Service (https://www.cas.org/support/documentation/chemical-substances). ^b^ Primary action of drugs according to the Dis-coveryProbe Library description. ^c^ Growth is expressed as percentage of the OD measured in the absence of drugs (0.1% DMSO) at 24 and 48 h post-initiation of the culture.

**Table 2:** Novel potential antiplague drugs.

Drug Name	Chemical Structure	Therapeutic Classification	IC_50_ (µM) (HIB ^1^)	IC_90_ (µM) (HIB)	IC_50_ (µM) (MHB ^2^)	IC_90_ (µM) (MHB)	MIC (µg/mL) (HIB)
Closantel	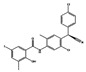	vermifuge	0.8	1.03	1.29	3	0.82
Closantel sodium	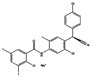	vermifuge	1.3	2.4	1.5	2.9	3.4
Bleomycin sulfate	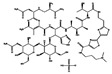	anticancer	2.2	3.77	2	2.54	15
Streptozocin	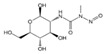	anticancer	2.2	4.75	2.65	5.36	2.6
Cetrimonium bromide	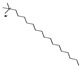	antiseptic	4.78	6.3	4.3	8.3	3.6
Cetylpyridinium chloride	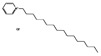	antiseptic	4.5	4.9	3.45	6.1	3.3
Chlorquinaldol	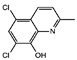	antiseptic	5.7	7	2.33	4	2.2
Zincpyrithione	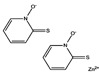	antifungal	3.29	4.8	2.89	4.49	1.588

^1^ Heart infusion broth (HIB). ^2^ Mueller−Hinton broth (MHB).

## Data Availability

Raw data is available upon request.
